# Synergistic Interaction of Low Salinity Stress With *Vibrio* Infection Causes Mass Mortalities in the Oyster by Inducing Host Microflora Imbalance and Immune Dysregulation

**DOI:** 10.3389/fimmu.2022.859975

**Published:** 2022-05-19

**Authors:** Xin Li, Ben Yang, Chenyu Shi, Hebing Wang, Ruihai Yu, Qi Li, Shikai Liu

**Affiliations:** ^1^ Key Laboratory of Mariculture, Ministry of Education, and College of Fisheries, Ocean University of China, Qingdao, China; ^2^ Laboratory for Marine Fisheries Science and Food Production Processes, Qingdao National Laboratory for Marine Science and Technology, Qingdao, China

**Keywords:** low salinity stress, *Crassostrea gigas*, *Vibrio alginolyticus*, digestive bacterial microbiota, immune response

## Abstract

A sudden drop in salinity following extreme precipitation events usually causes mass mortality of oysters exposed to pathogens in ocean environment. While how low salinity stress interacts with pathogens to cause mass mortality remains obscure. In this study, we performed an experiment by low salinity stress and pathogen infection with *Vibrio alginolyticus* to investigate their synergistic effect on the mortality of the Pacific oyster toward understanding of the interaction among environment, host, and pathogen. We showed that low salinity stress did not significantly affect proliferation and virulence of *V. alginolyticus*, but significantly altered microbial composition and immune response of infected oysters. Microbial community profiling by 16S rRNA amplicon sequencing revealed disrupted homeostasis of digestive bacterial microbiota with the abundance of several pathogenic bacteria being increased, which may affect the pathogenesis in infected oysters. Transcriptome profiling of infected oysters revealed that a large number of genes associated with apoptosis and inflammation were significantly upregulated under low salinity, suggesting that low salinity stress may have triggered immune dysregulation in infected oysters. Our results suggest that host-pathogen interactions are strongly affected by low salinity stress, which is of great significance for assessing future environmental risk of pathogenic diseases, decoding the interaction among environment, host genetics and commensal microbes, and disease surveillance in the oyster.

## Introduction

Due to global warming, ice caps and glaciers melting (1), extreme precipitation events are likely to occur more frequently (2, 3), which poses increasing threats to marine ecosystems of coasts and estuaries (4–6). As an important marine economic shellfish species, the oysters inhabit in estuaries and intertidal areas. Drastic changes in the salinity of estuaries and intertidal areas caused by heavy rainfall have a great effect on growth and reproduction of oysters (7, 8). The fluctuations of salinity can cause physiological stress (9), promote pathogenesis of microbial infection, and aggravate disease outbreaks (10–12). As a benthic filter-feeders, oysters are at risk of exposure to tremendous pathogenic microorganisms, such as *Ostreid herpesvirus 1* (*OsHV-1*), *Vibrio parahaemolyticus*, *V. harveyi* and *V. alginolyticus* (13–16). Disease outbreaks caused by pathogenic microbes pose a major threat to marine ecosystem and shellfish aquaculture in many countries.

Different Vibrio species produce a series of extracellular products, which are potential pathogenic factors for marine animals. These toxins mainly include extracellular hemolysin, protease, lipase, siderophore, exopolysaccharide and effectors delivered *via* the type III secretion systems (17–21). Salinity can regulate the progression of diseases by affecting pathogens, hosts, or host-pathogen interactions. It has been reported that changes in salinity may have important effects on *OsHV-1* transmission in oysters (22, 23). Understanding the interaction among environment, host, and pathogens is essential for exploring the complex aetiology of oyster disease caused by abiotic and biotic stress.

Alteration of the gut microbiota is involved in animal pathology (24). Studies have shown that the dynamic changes of the microbiota are closely related to the development, health, metabolism and immunity of the host (25, 26). The digestive flora of many aquatic organisms not only promotes the body’s nutrient absorption by secreting digestive enzymes, but also protects the body against the invasion of pathogens (27). However, studies have also shown that the change of host’s external environmental conditions or pathogen invasion may cause the imbalance of host’s internal microbial homeostasis, thereby, symbiotic bacteria may transform into opportunistic pathogens to mediate the development of host diseases. For instance, opportunistic bacteria are involved in secondary bacterial infections in oysters that are immune-compromised due to viral infection (28). Gut microbiota may have changed before invertebrate disease, providing indicators of host health (29). Studies have shown that the disrupted microbial community structure was observed in the hemolymph of diseased oysters affected by temperature, and the increased abundance of potential pathogenic bacteria such as Arcobacter spp. may be related to the high mortality of oysters (30). In addition, it has also been reported that *Mycoplasma* was the dominant flora in the oyster gut when oysters were exposed to ocean acidification, which may have adversely affected intestinal health of oysters (31). Therefore, the stability of the microbial community is critical to health and viability of the host (32).

Transcriptome profiling has been widely used to analyze various traits, including heat stress (33), salinity stress (34, 35), and viral infections (36), revealing differentially expressed genes related to various immune pathways, including macrophage (37), interleukin-1 receptor-associated kinase (38), C1q complement system (39), apoptosis pathway (40), and the NF-κB, toll-like receptor and MAPK signaling pathways (41). Among which, inflammatory factors and apoptosis play an important role in innate immune response. Pro-inflammatory mediators and oxidative stress can induce cell apoptosis (42–44). The apoptosis or necrotic cell death may also cause inflammation, however, if the inflammatory response is excessively severe, there is danger of continuous tissue damage, forming an auto-amplification loop that causes organ damage (43, 45). There is growing evidence that the gill tissue of oysters produces blood cells and is the potential hematopoietic organ. When oysters are attacked by pathogens, the gill becomes the main tissue to initiate immune response and promote the proliferation of immune-related cells (46, 47). Numerous studies have been conducted on the immune mechanism in oyster by transcriptome profiling of gill tissue (48–50). Studies have shown that sharp changes in salinity, when combined with additional co-occurring stressors, appeared to trigger dysregulation of the immune response in fish, potentially making organisms more susceptible to infectious diseases (51).

Disease outbreaks in the marine environment depends on interaction among the host, pathogens and environmental factors (52). However, most studies only showed that the host response to single environmental factor, making it difficult to characterize diseases with complex etiology. Thus, understanding of diseases caused by interaction among the host, environment and pathogens remains obscure. In a previous study, we identified *V. alginolyticus* as a causative pathogen associated with mass summer mortality of the Pacific oyster (*Crassostrea gigas*), an important oyster species for aquaculture in China (16). In the present work, we performed an experiment by mimicking a sudden drop of salinity and pathogen infection with *V. alginolyticus* to investigate their synergistic effect on the mortality of the infected oysters. We tested the effects of salinity on the growth and virulence of *V. alginolyticus*, and also investigated the digestive bacterial microbiota dynamics and transcriptome responses of infected oysters. The results showed that high mortality rate of infected oysters under low salinity stress was caused by the destruction of the bacterial balance and immune homeostasis, which suppressed the host resistance to *V. alginolyticus*. This work has great implications for decoding mechanisms of synergistic interactions among environmental conditions, host physiology, and pathogen, and providing insights into assessing future environmental risk of pathogenic diseases.

## Materials and Methods

### Experiment Animals

Healthy *C. gigas* (average wet weight of 18.2 ± 2.8 g) obtained from an oyster farm (salinity of 30‰) in Rongcheng (Shandong, China) were used for experiment. The oysters were transported to laboratory and acclimatized in 30‰ seawater for two weeks. Continuous aeration was provided and the water quality was monitored. The oysters were fed with concentrated Chlorella vulgaris ad libitum. The water quality parameters during experimental challenge trials were as follows: pH at 8.1-8.2, dissolved oxygen at 8.0-9.0 ppm, salinity at 30‰, and nitrite at 1-3 ppm. Unused feed and faecal matter were cleaned up daily and 25% water was changed every other day. Experiments related to *C. gigas* were carried out in strict accordance with the Management Rule of Laboratory Animals (Chinese Order No. 676 of the State Council, revised 1 March 2017). The Committee on the Ethics of Animal Experiments of Ocean University of China has approved the relevant experimental procedures.

### Salinity Stress and *V. alginolyticus* Infection

Oysters were randomly assigned to glass tanks containing 20L seawater. The pathogenic *V. alginolyticus* strain isolated and purified previously (16) was inoculated in 50 mL 2216E broth. The 5×10^8^ CFU mL^-1^ bacterial suspension was used for infection. The infection method was used to infect oysters in this work as previously described (53–56). The *C. gigas* were anesthetized with magnesium chloride (MgCl_2_, 50 g/L) solution before being injected with *V. alginolyticus*. Each oyster was intramuscular injected with 50 μL (5×10^8^ CFU mL^-1^) bacterial suspension volume through a microsyringe (100 ± 0.5 μL) according to our pre-trial results and previous reports (30, 57). After injection, infected oysters were placed in three 20L glass tanks containing 10‰ seawater (defined as “10‰Vibrio” group) or 30‰ seawater (defined as “30‰Vibrio” group), respectively. The experiment was performed in triplicate with 30 individuals in each tank. The low salinity (10‰ or 20‰) of seawater used in this work was prepared by mixing tap water fully aerated for 24h and seawater prepared by natural sand filtration. Oysters injected with an equal volume of artificial seawater and cultured in 30‰ seawater were used as control. During the experiment, digestive gland tissues of *C. gigas* were sampled at 0, 12, 48 and 72h post infection for bacterial flora analysis. Gill tissues were sampled at 0, 12, and 48h post infection for transcriptomic analysis. During sampling, three individuals from each tank were randomly selected at each time point for dissection of tissues including gill and digestive gland. For each tissue, equal amount dissected from the three individuals were pooled and stored in liquid nitrogen which was used as one biological replicate. In total, three biological replicates were obtained for each group at each time point.

### Effect of Low Salinity on Growth and Virulence of *V. alginolyticus*

The *V. alginolyticus* was cultured in Zobell Marine Broth 2216E at 28 °C for examination of growth and virulence. *V. alginolyticus* was inoculated in 50 mL flask containing 10 mL tryptone soy broth in different concentrations of sterile saline (10‰, 20‰ and 30‰) for bacterial growth test at 28 ± 1°C. Bacterial growth at 12, 24, 48, 72, and 96h was obtained by measuring optical density (OD) at 600 nm. Each experiment was repeated three times.

For the determination of extracellular enzyme activity of *V. alginolyticus*, overnight cultures of the bacterial strain in tryptone soy broth media in different concentrations of sterile saline (10‰, 20‰ and 30‰) were diluted to an OD 600 of 0.5. Then, 10 μL of the diluted cultures was spotted in the middle of the test plates. All assays were carried out in triplicate. In this assays, 2216E agar plates supplemented with 0.5% starch, 1% Tween 80, or 1% egg yolk emulsion (58), respectively, were used. The development of colorless and transparent circle around the colonies was observed for amylase after dripping Lugol’s iodine solution. The diameter of the opalescent zones formed around *V. alginolyticus* colonies by lipase and phospholipase was measured after 2-4 days.

For determination of virulence of *V. alginolyticus* in *C. gigas*, *V. alginolyticus* was cultured in tryptone soy broth media in different concentrations of sterile saline (10‰, 20‰ and 30‰) for 24 hours. The *V. alginolyticus* pellet was obtained by centrifugation at 8000 × g for 5 minutes, which was then resuspended in saline to obtain 5 × 10^8^ CFU mL^-1^ bacterial suspension for injection. Ten oysters were used for each of the treatment (10‰, 20‰) and control groups (30‰). The experimental infection was done in triplicate. Each oyster was injected with a total of 50 μL bacterial suspension into the adductor muscle. After injection, oysters were placed in 20 L glass tanks containing 30‰ seawater, and observed for one week. Control oysters were injected with 50 μL sterile saline solution. Differences between treatment groups were compared using a multiple comparisons (Tukey) test.

### Bacterial Microbiota Analysis

Total DNA was isolated from digestive gland samples using the E.Z.N.A.^®^ soil DNA kit (Omega Bio-tek, Norcross, GA, USA). The genomic DNA quality was confirmed by running 1% agarose gel electrophoresis. PCR was performed using two universal bacterial primers 341F (5’-CCTAYGGGRBGCASCAG-3’) and 806R (5’-GGACTACNNGGGTATCTAAT-3’). The PCR amplification was carried out in triplicate as follows: a first step of 3 min at 95°C, followed by a second step of 27 cycles consisting of 95°C for 30 s, 55°C for 30 s, 72°C for 45 s, and finally a single extension at 72°C for 10 min. After the amplification product was obtained from 2% agarose gel, it was purified according to the instructions of the AxyPrep DNA Gel Extraction Kit (Axygen Biosciences, Union City, CA, USA). Quantification was performed using the QuantiFluor^™^-ST fluorescent quantitative system (Promega, USA). Sequencing was performed using an Illumina MiSeq platform (Illumina, San Diego, USA) for 300 bp paired end reads.

Fastp (version 0.20.0) software evaluated the quality of the original fastq format files. OTUs were determined by UPARSE software (version 7.1) with similarity threshold of 97% (59). The RDP classifier analyzed the species taxonomy of each sequence against the Silva 16S rRNA database, and the confidence threshold was set to 70% (60). Rarefaction curve were analyzed by Mothur software (version 1.30.2). Significant differences of alpha diversity index (Shannon and Chao I) among groups were calculated with one-way ANOVA. *P* < 0.05 was considered statistically significant. PCoA was conducted according to the Bray-Curtis distance matrix calculated. The gplot package of R software computed a heat map of the relative abundance of flora.

### Transcriptome Profiling of Infected Oysters

The gill samples of two salinity groups (“10‰Vibrio” and “30‰Vibrio”) were used for transcriptome analysis. RNA was extracted from *C. gigas* gill tissue using Trizol reagent (Invitrogen). High quality RNA had three clear bands after being confirmed by 1% agarose gel electrophoresis, and then RNA concentration measured by NanoDrop (Thermo Fisher Scientific) was at least 200 ng/μL and the ratio of OD260/280 was 1.8~2.2. The RNA Nano 6000 Assay Kit detects an RNA Integrity Number (RIN) value of at least 7 to meet library construction requirements for the NEBNext^®^ Ultra^™^ RNA Library Prep Kit. Qualified RNA was enriched using poly-T oligo-attached magnetic beads, and then the obtained mRNA was fragmented into small fragments by NEB fragmentation Buffer. cDNA synthesis was performed using fragmented mRNA as template, and random hexamers and RNase H- were added. The synthesis of the second strand is based on dNTP as a substrate, and RNase H and DNA polymerase I are added at the same time. The purified cDNA was end-repaired, 3’-adenylated, and adaptor ligated, and cDNA fragments were selected using AMPure XP microspheres. After PCR enrichment, the qualified final libraries were sequenced using the Illumina high-throughput sequencing platform NovaSeq 6000.

The quality of raw data obtained by sequencing was first assessed by FastQC, and then the sequencing data was trimmed, mainly including removing reads with adapters, low quality, and N-containing reads. High quality clean reads for subsequent analysis are finally obtained. The clean reads after quality control are accurately and quickly aligned to the indexed reference genome (GCA_902806645.1) by Hisat2 (v2.2.1) (61, 62), and the alignment rate is higher than 70%. The counts of reads mapped to each gene were obtained using FeatureCounts (v1.6.0) (63). The expression levels of each gene in different salinity treatment groups were calculated using Fragments per Kilobase of transcript per million mapped reads (FPKM) (64). The differential gene expression of “10‰Vibrio” and “30‰Vibrio” groups at different time points was analyzed by DEseq2 (1.30.1). P-values derived from DEseq2 analysis were adjusted using the method of Benjamini and Hochberg. Significantly differentially expressed genes (DEGs) were screened with an adjusted P-value < 0.05 and |fold-change| > 1. These differential genes were then subjected to GO enrichment analysis and KEGG pathway analysis, and the adjusted P value of each functional pathway less than 0.05 was considered to be significantly enriched.

### Quantitative Real-Time PCR Analysis

We performed qRT-PCR analysis of genes used for immune-inflammatory response and transcriptomic data validation. Specific primers of related genes are shown in **Supplementary Table 1**. *EF-1α* gene is an internal reference gene for gene expression in gill tissues. The qRT-PCR experimental system was configured according to SYBR^®^ Premix Ex Taq^™^ (TaKaRa) and then performed on the LightCycler 480 real-time PCR instrument (Roche Diagnostics, Burgess Hill, UK). The reaction conditions were as follows: an initial step of 5 min at 95°C; followed by 40 cycles of 5 s at 95°C, 30 s at 58°C and 30 s at 72°C. The specific amplification was determined by solubility curves. The relative expression levels of genes were calculated using 2^−ΔΔCt^ method (65).

### Statistical Analysis

Survival curves were used to analyze the mortality dynamics of infected oysters under different salinity conditions. The log rank test was used to compare survival rate among groups. All data in *V. alginolyticus* growth and virulence assay experiments were expressed as mean ± standard error (S.E.), and multiple comparison (Tukey) tests were used to compare the differences among groups. We used one-way ANOVA to compare differences in the alpha diversity index of microbial communities between salinity treatment groups. The PCoA was calculated using the Bray-Curtis distance matrix to compare differences among groups. For all statistical analyses, differences were considered significant when *P* < 0.05.

## Results

### Oyster Survival Analysis

**Figure 1:**
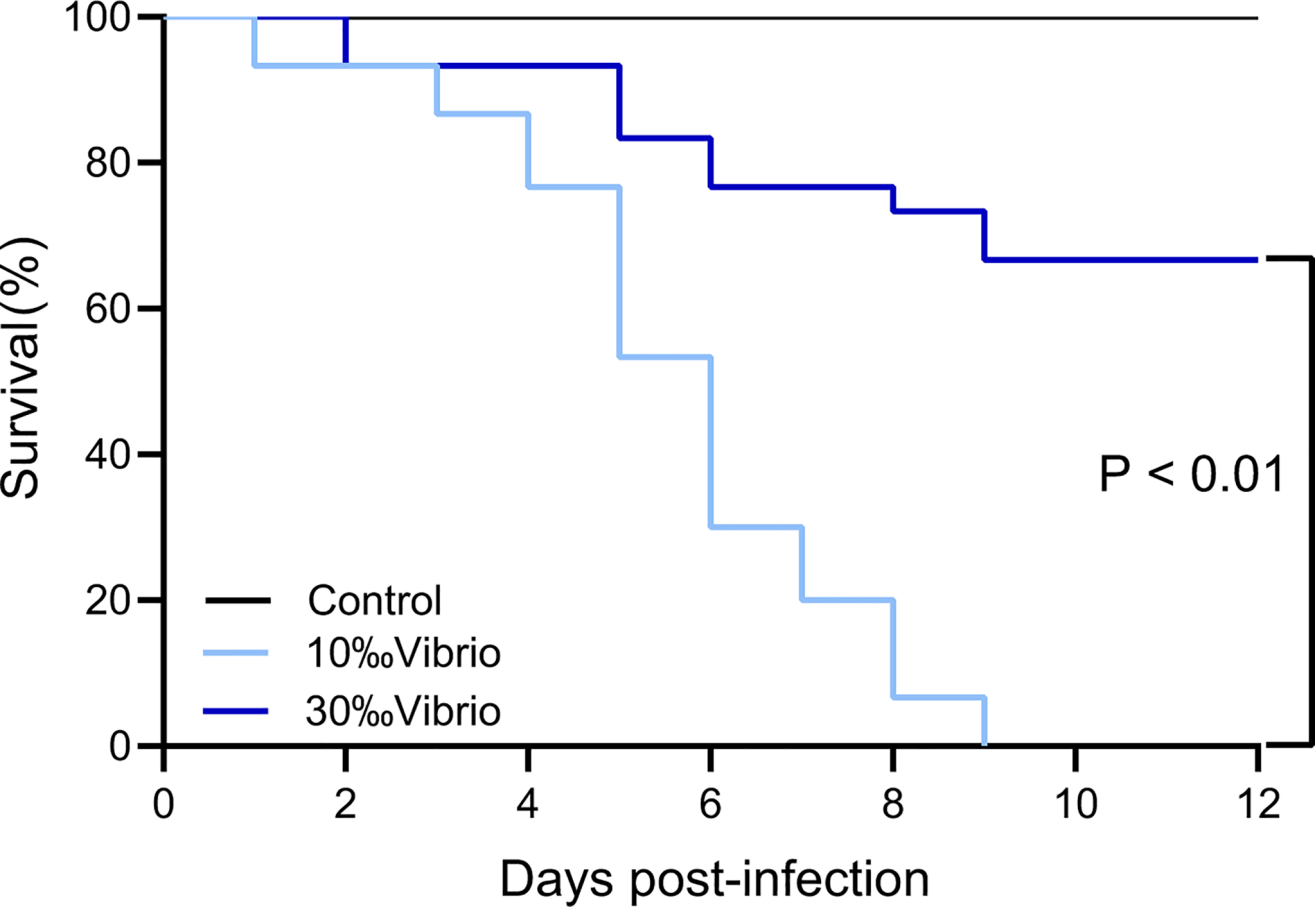
Survival analysis of C. gigas infected with *V. alginolyticus* at different levels of salinity. The mortality of *C. gigas* in each salinity group (in triplicate) was monitored for 12 days post infection with *V. alginolyticus*. *P* < 0.01, log rank test; n = 30.

The mortality of *C. gigas* infected with *V. alginolyticus* at normal salinity (30‰) and low salinity (10‰) conditions was monitored throughout the disease progression (**Figure 1**). Through a 12-day experiment, the infected oysters at normal salinity (30‰) showed 33% mortality, while oysters at low salinity (10‰) showed 100% mortality. No mortality was observed in the control group oysters injected with artificial seawater. The observation suggested that low salinity stress had a significant effect on the pathogenesis of *V. alginolyticus* to cause mortality of the oysters.

### Effect of Low Salinity on the Growth and Virulence of *V. alginolyticus*

**Figure 2:**
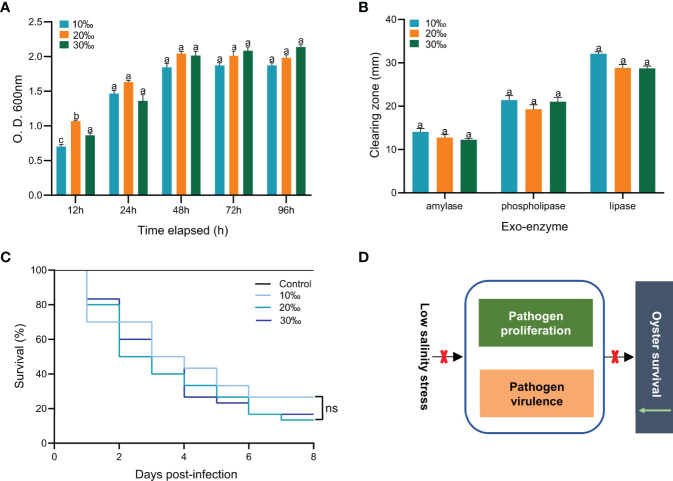
Effect of low salinity on growth and virulence of *V. alginolyticus*. **(A)** Effect of salinity on growth of *V. alginolyticus*. Bacterial amounts were determined at 12, 24, 48, 72 and 96 h. Each bar represents the mean ( ± S.E.) of three determinations. The same letter at the same time point indicates that the difference is not significant. **(B)** Effect of salinity on amylase, lipase and lecithinase activities of *V. alginolyticus*. Each bar represents the mean ( ± S.E.) of three determinations. The same letter at the same time point indicates that the difference is not significant. **(C)** Survival analysis of oysters infected with *V. alginolyticus* cultured with different levels of salinity. n=30, in triplicate; ns, not significant. **(D)** The high mortality of oysters caused by low salinity is not achieved by affecting the growth and virulence of *V. alginolyticus*.

To explore the effect of low salinity on *V. alginolyticus* infection in the *C. gigas*, we examined the growth and virulence of *V. alginolyticus* under different levels of salinity (**Figure 2**). No significant difference was observed for growth of *V. alginolyticus* cultured with different levels of salinity (10‰, 20‰ and 30‰) after 12h (*P* > 0.05) (**Figure 2A**). Further examination of the activities of amylase, lipase and lecithinase of *V. alginolyticus* cultured with different salinity (10‰, 20‰ and 30‰) showed no significant difference either (*P* > 0.05) (**Figure 2B**). To further determine the effect of low salinity on the virulence of *V. alginolyticus* in infected oysters, we injected *V. alginolyticus* cultured with different levels of salinity (10‰, 20‰ and 30‰) into oysters and observed the mortality (**Figure 2C**). The oyster death was first observed on one day post injection. Cumulative mortality of *C. gigas* at the end of the experiment was not significantly different among the three groups of different salinity (10‰, 20‰ and 30‰) (*P* > 0.05). Together, these results showed that changes in salinity level did not have a significant effect on growth and virulence of *V. alginolyticus* (**Figure 2D**). Therefore, the effect of low salinity on the oyster’s mortality may not be caused by affecting *V. alginolyticus*, but the oysters.

### Effect of Low Salinity on Digestive Bacterial Microbiota of the Oysters

**Figure 3:**
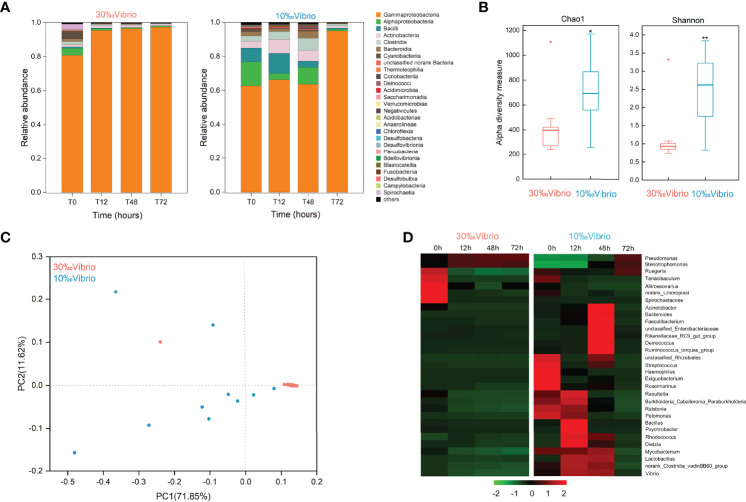
Microflora changes in *V. alginolyticus* infected oysters exposed to salinity of 10‰ and 30‰. **(A)** Relative proportion of bacteria (class level) for “10‰Vibrio” and “30‰Vibrio” oysters. T0, T12, T48 and T72 indicated different sampling time-points (in hours) during the experiment. **(B)** Temporal dynamics of alpha diversity in *V. alginolyticus* infected oysters at different salinity (10‰ and 30‰). Chao1 and Shannon’s H index for “30‰Vibrio” and “10‰Vibrio” oysters. Asterisk indicated *P* < 0.05. **(C)** Principal coordinate analysis (PCoA) plot of the Bray-Curtis dissimilarity matrix of the microflora comparing “10‰Vibrio” and “30‰Vibrio” oysters. The axes of the PCoA represent the two synthetic variables that explained the most variation of the dataset. **(D)** The abundance of each bacteria at the genus level in “10‰Vibrio” and “30‰Vibrio” oysters. Only genera with a relative proportion >0.5% in at least one sample are shown. The abundance of each type of bacteria in “10‰Vibrio” and “30‰Vibrio” oysters at the genus level during the *V. alginolyticus* infection. Green colour (smaller Log10 index) indicated lower abundance. Red colour (larger Log10 index) represented higher abundance.

To investigate the microbiota dynamics of *C. gigas* infected with *V. alginolyticus* under normal salinity (denoted as “30‰Vibrio” hereafter) or low salinity (denoted as “10‰Vibrio” hereafter), we analyzed digestive bacterial microbiota using 16S rRNA amplicon sequencing. Overall, we obtained 1,518,721 raw sequencing reads from 24 samples (2 salinity gradients, 4 sampling times, in triplicate, **Supplementary Table 2**). The number of OTUs from all samples was 3475. The reliability of the sequencing data was confirmed by the species richness rarefaction curve (**Supplementary Figure 1**). The dominant class of intestinal microbiota in infected oysters was *Gammaproteobacteria*. The abundance ratio of *Gammaproteobacteria* tended to be stable at normal salinity (both greater than 80%), while at low salinity, it increased from 62.69% at 0h to 95.07% at 72h. During the whole experiment, the changes of microbiota composition in “10‰Vibrio” oysters were more significant than that of “30‰Vibrio” oysters (**Figure 3A**). There were 27 genera with relative abundance higher than 0.1% in at least one sample, and the remaining genera were grouped as “Others”. The Chao1 and Shannon’s H indexes of alpha diversity increased significantly in “10‰Vibrio” oysters in comparison with “30‰Vibrio” oysters (Chao1: df = 22; P = 0.010 and Shannon’s H index: df = 22; P =0.001) (**Figure 3B**). Consistently, the principal coordinate analysis (PCoA) of the Bray-Curtis dissimilarity matrix (beta diversity) revealed a higher microbiota dispersion in “10‰Vibrio” oysters than in “30‰Vibrio” oysters (F = 2.330, R^2^ = 0.505; P = 0.035; PERMANOVA) (**Figure 3C**). The hierarchical clustering heat map analysis at the genus level also showed that the microbial community structure of “10‰Vibrio” and “30‰Vibrio” was significantly different during infection of *V. alginolyticus* (**Figure 3D**). Apparently, compared with “30‰Vibrio” oysters, bacteria associated with marine organism diseases including *Vibrio*, *Acinetobacter*, *Bacteroides* and *Streptococcus* became dominant genus in “10‰Vibrio” oysters during the process of infection.

### Effect of Low Salinity on Immune Response of the Oysters

**Figure 4:**
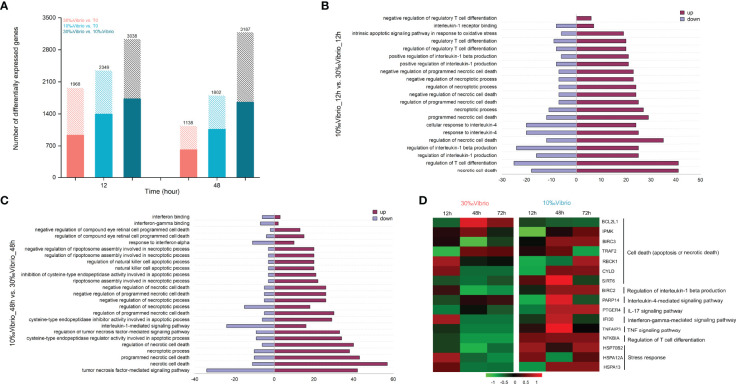
Comparative transcriptome profiling of *V. alginolyticus* infected oysters exposed to salinity of 10‰ and 30‰. **(A)** Identification of differentially expressed genes between *V. alginolyticus* infected oysters exposed to normal salinity (30‰) and low salinity (10‰) at 12h and 48h post infection. The filled colored bars indicated number of genes that were expressed at higher levels, while hashed bars indicated number of genes expressed at lower levels. **(B)** GO categories associated with necrotic cell apoptosis or death and inflammation that were significantly enriched at 12h. The red-colored bars indicated number of genes that were expressed at higher levels in 10‰Vibrio oysters, while purple-colored bars indicated number of genes expressed at higher levels in 30‰Vibrio oysters. **(C)** GO categories associated with necrotic cell apoptosis or death and inflammation that were significantly enriched at 48h. The red-colored bars indicated number of genes that were expressed at higher levels in 10‰Vibrio oysters, while purple-colored bars indicated number of genes expressed at higher levels in 30‰Vibrio oysters. **(D)** Verification of expression profiles of genes associated with necrotic cell apoptosis or death and inflammatory response. Differential expression analysis was performed between each time point and time zero. The intensity of the color from green to red indicates the magnitude of differential expression in log_2_(foldchange).

To determine how low salinity affects the host immune response to *V. alginolyticus*, we compared transcriptional profiles of *C. gigas* infected with *V. alginolyticus* at low salinity and normal salinity using RNA-seq. Total RNA of gill tissues collected from 0h, 12h and 48h (denoted as T0, T12 and T48, respectively) post infection at different salinity (10‰ and 30‰) were sequenced, with 73.9-82.5% of reads being mapped to the *C. gigas* reference genome (**Supplementary Table 3**). Validation by RT-qPCR confirmed the results of the mRNA sequencing (**Supplementary Figure 2**, **Supplementary Table 1**). Differentially expressed genes (DEGs) between each time point and the T0 control (“30‰Vibrio” versus T0, “10‰Vibrio” versus T0 at 12h and 48h post infection), and between the two groups (“30‰Vibrio” versus “10‰Vibrio” at 12h and 48h post infection) were identified (**Figure 4A**). Comparison between each salinity group and T0 (“30‰Vibrio” versus T0 and “10‰Vibrio” versus T0) showed that the number of DEGs in infected oysters at low salinity was higher than that at normal salinity (**Figure 4A**), suggesting that low salinity stress posed additional transcriptome alternations in addition with *V. alginolyticus* infection. To further clarify the specific factors that cause phenotypic differences in survival post infection under low salinity, we compared the two groups (“30‰Vibrio” versus “10‰Vibrio”), and identified a total of 3038 and 3187 DEGs in “10‰Vibrio” oysters compared with “30‰Vibrio” oysters at 12h and 48h post infection, respectively (**Figure 4A**, **Supplementary Table 4**). To infer the biological processes regulated at low salinity, we performed gene ontology (GO) enrichment analysis (*P* < 0.05) (**Supplementary Table 5**). The results showed that large numbers of DEGs were more enriched in functional categories related to immunity (26% at 12h; 27.8% at 48h) **(** **Supplementary Table 5** **)**. Interestingly, the majority of functional categories related to immunity were associated with inflammatory cytokines and apoptosis or cell death functional pathways (55% at 12h; 35.2% at 48h) (**Supplementary Table 5**), and most of genes involved in these pathways were induced in “10‰Vibrio” oysters (**Figures 4B, C**), such as “necrotic cell death”, “regulation of interleukin-1 beta production”, “interleukin-4-mediated signaling pathway”, “regulatory T cell differentiation”, and “intrinsic apoptotic signaling pathway in response to oxidative stress”. KEGG enrichment analysis of DEGs enabled further identification of significantly enriched pathways (*P* < 0.05) (**Supplementary Table 6**), including inflammatory pathways such as the “NF-Kappa B signaling pathway” and “TNF signaling pathway”. In addition, the “apoptosis”, “necroptosis”, “Nod-like receptor signaling pathway”, “IL-17 signaling pathway”, “toll-like receptor signaling pathway” and “TH17 cell differentiation” were also enriched at 12h post infection. Taken together, we posited that the infected oysters had severely inflammatory response at low salinity compared with normal salinity, which were further verified by selection of representative genes. Sixteen DEGs related to cell death (apoptosis or necrotic death) and inflammation were chosen to verify the expression pattern between “30‰Vibrio” and “10‰Vibrio” (**Figure 4D**, **Supplementary Table 1**). The results showed that genes related to cell death (apoptosis or necrotic death) (*BIRC3*, *TRAF2*, *CYLD*, *SIRT6*), regulation of interleukin-1 beta production (*BIRC2*), and interleukin-4-mediated signaling pathway (*PARP14*), IL-17 signaling pathway (*PTGER4*), interferon-gamma-mediated signaling pathway (*IFI30*), TNF signaling pathway (*TNFaip3*), regulation of T cell differentiation (*NFKBIA*) and oxidative stress (*HSP70B2*, *HSPA12A*, and *HSPA13*) were expressed at higher levels in 48h of “10‰Vibrio” oysters. Consistently, expression of *BCL2L1* gene, which inhibits cell death, was suppressed in 48h of “10‰Vibrio” oysters. In summary, these results suggest that low salinity stress induced more intense inflammatory and cell death (apoptosis or necrotic death) responses in *V. alginolyticus* infected oysters at 48h. In addition, the results also showed that a few genes associated with stimulation of apoptosis and inflammation (*TRAF2*, *SIRT6*, *PARP14*, *IFI30, TNFaip3*) were expressed at low levels at 72h in the “10‰Vibrio” oysters, while most genes were expressed at high levels. The results showed that the intensity of the inflammatory response decreased slightly in the later stage, but the inflammatory response still existed at 72h.

## Discussion

When experiencing extreme weather in the context of disease outbreaks caused by pathogenic microbes, it may seriously threaten the economic and ecological functions of coastal region marine organisms. Therefore, it is important to investigate the pathological processes that might be affected by extreme precipitation events and pathogen challenges. In this study, we observed the high mortality of *C. gigas* infected with *V. alginolyticus* at low salinity, and performed further investigation on their synergistic effect on the mortality of the infected oysters. The mortality rate of infected oysters exposed to low salinity (10‰) was 100%, in contrast that the mortality rate of infected oysters exposed to normal salinity (30‰) was 33%. The results showed that low salinity stress could trigger an increased mortality in oysters infected with pathogens, which is consistent with previous studies that reported a sharp increase in mortality of oysters exposed to extreme precipitation events (66). We further investigated the cause for mass mortality of infected oysters at low salinity (“10‰Vibrio”) and speculated whether low salinity stress has an effect on pathogen infection or host physiology, or both.

Changes in the natural environment affect the growth of pathogens and their secretion of virulence factors. Before the host’s immune defense system fully functions, fast-growing pathogens may secrete more virulence factors to destroy the host’s non-specific defense and lead to occurrence of disease (67). In addition, in our study, the change of salinity had no significant effect on the growth and virulence of *V. alginolyticus*. This suggested that high mortality rate observed in “10‰Vibrio” oysters may not be caused by affecting growth and virulence of *V. alginolyticus*.

To better understand the relationship between the high mortality of infected oysters at low salinity and intestinal microbiota composition, bacterial communities in the digestive gland of oysters were examined. Our results showed that alterations in microbiota composition and diversity in “10‰Vibrio” oysters were more significant than that in “30‰Vibrio” oysters. Previous studies have reported that salinity stress and pathogen exposure can cause changes in intestinal microbiota composition of various aquatic organisms (28, 68–70), while the occurrence of many diseases is associated with changes in intestinal microbial composition (71). Intestinal microorganism can provide protection against pathogens by producing inhibitory compounds and competing for nutrients and space. Therefore, we concluded that such changes in the intestinal microbiome caused by low salinity might exacerbate disease progression in infected oysters.

The finding of *Gammaproteobacteria* as the dominant class of intestinal microbiota in infected oysters is consistent with reports in previous studies (28, 68, 72). Low salinity stress affected the relative abundance of *Gammaproteobacteria* in the oyster bacterial community. The OTUs with increased abundance in “10‰Vibrio” oysters were assigned to the genera *Vibrio*, *Acinetobacter*, *Bacteroides*, and *Streptococcus*, all of which have been previously associated with diseases in some aquatic organisms. There are growing studies revealed the role of *Vibrio* communities in oyster disease outbreaks (13, 73–76). Previous studies had shown that the original *Vibrio* community in oysters had been replaced by pathogenic *Vibrio* before the disease outbreaks (56), especially when oysters were exposed to stressors that may facilitate the transition to more pathogen-dominant communities (28, 74). Our work also suggested the increased abundance of *Vibrio* community when infected oysters were exposed to low salinity. Although the relative abundance was low, it could have a significant influence on the host health (77). In addition, *Acinetobacter* (78), *Bacteroides* (79–81), and *Streptococcus* (82–84) were over-represented in “10‰Vibrio” oysters, which have been identified as known pathogens in fish or crabs. Low salinity stress may disrupt the homeostasis of the microbial composition in infected oysters, likely leading to proliferation of various opportunistic pathogens and inflammation in oysters. The stable microbiome of “30‰Vibrio” may suggest their potential role in host adaptation to stressors (85–88).

Comparative expression profiling of Vibrio infected oysters at low and normal salinity provided insights into molecular basis associated with high mortality of oysters under biotic and abiotic stresses. Functional analysis of DEGs revealed that gene pathways related to necrotic cell apoptosis or death, interleukin-1 production, interleukin-4 mediated signaling pathway, and T cell differentiation were significantly enriched. Interestingly, most of the genes involved in these significantly enriched functional pathways were upregulated upon low salinity stress. It has been reported that necrotic cell apoptosis or death is a critical determinant for the initiation of inflammation, and damage-associated molecular patter (DAMP) signals sent by dead cells can attract more monocytes, inducing a vicious cycle of inflammation and accelerating disease development (89). Interleukin-1 (IL-1) family cytokines IL-1β play key roles in inflammation (90), and its excessive and/or dysregulated activity can cause common inflammatory disorders (91, 92). Naive CD4^+^ T cells can be differentiated into Th17 cells that produce IL-17, Th1 cells that produce TNF-α and IFN-γ, and Th2 cells that produce IL-4, and during intracellular bacterial infection (93, 94). The excessive cytokines may induce an imbalance in the Th1/Th2 ratio, thereby enhancing immune activation and inflammatory response, which is closely related to the onset and severity of colitis, inflammatory bowel disease (IBD) and asthma (95).

Inflammation protects the host from pathogens and can repair damaged tissues. However, excessive inflammation can cause tissue damage and malaise (96). We further validated expression profiles of critical genes involved in cell death (apoptosis or necrotic death), inflammatory factors and oxidative stress. We showed that the majority of these genes were expressed at higher levels in infected oysters exposed to low salinity stress. These results were consistent with the results of high expression of apoptosis and inflammatory cytokine related genes in immune-damaged fish and chickens (51, 97–99). Furthermore, an auto-amplification loop leading organ damage may be the result of the interaction of apoptosis and inflammatory responses (45).

Functional analysis of DEGs also showed that some immune functions (i.e. defense response to bacteria, complement activity, antimicrobial peptide biosynthesis, antigen processing and presentation) were significantly enriched in infected oysters at low salinity. Nevertheless, as shown by the high mortality of infected oysters at low salinity, the increase of immune-related factors does not necessarily indicate that the organism’s resistance to microbial disease is high. These results suggest that low salinity stress induces more severe inflammatory and apoptotic responses in infected oysters, and these factors may interact to cause immune damage and disrupt basal immune homeostasis, similarly as the phenomena that have been reported in fish (51, 100). Low salinity creates unfavorable environment for effective immune defense against pathogen infection in oysters. However, mechanism by which low salinity induces immune damage in infected oysters deserves further investigations.

**Figure 5:**
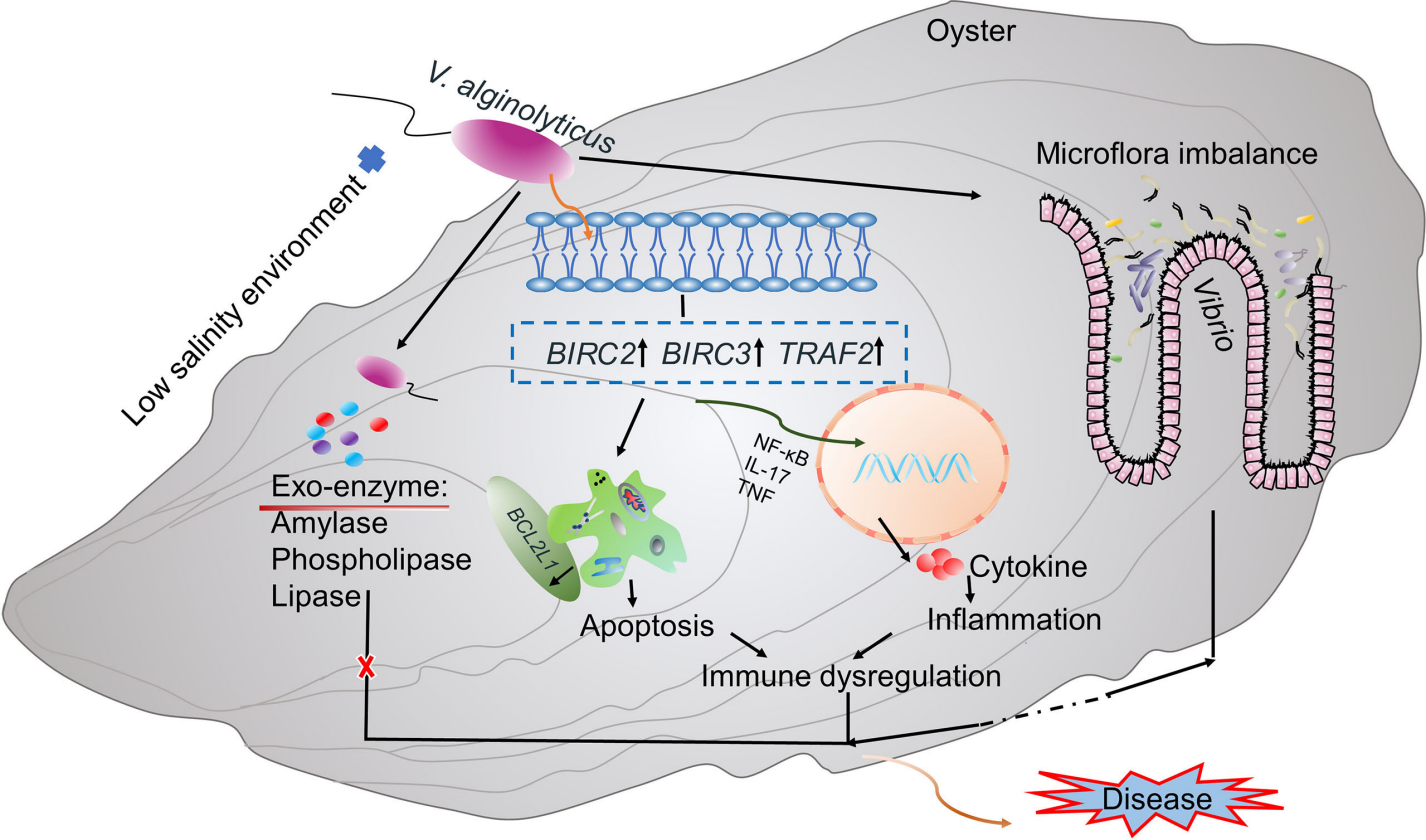
A proposed model for synergistic interaction of low salinity stress, *Vibrio* infection and host response. Microflora imbalance and immune dysregulation caused by low salinity stress are hypothesized to cause high mortality of oysters infected with *V. alginolyticus* in low salinity environment.

Regulation of immune homeostasis and stable intestinal flora are important for maintaining host health (101). Our final results showed that low salinity environment causes changes in digestive bacterial microbiota of infected oysters, leading to increased abundance of pathogenic bacteria such as *Vibrio* and disruption of microflora homeostasis of the host. Moreover, the low salinity stress induced alterations of expression of genes involved in apoptosis and inflammation (e.g, *BIRC2*, *BIRC3*, *TRAF2*), which promotes production of inflammation-related cytokines that lead to immune dysregulation in oysters. Together, microflora imbalance and immune dysregulation caused by low salinity stress drive high mortality of the oysters (**Figure 5**). The increased abundance of digestive gland pathogens and the enhanced immune inflammatory response were more significant at 48h of the experiment. In addition, we also found that a few genes related to immune inflammation and apoptosis were expressed at low levels at 72h, indicating that the intensity of the inflammatory response decreased slightly, which may be related to the homeostasis recovery of the digestive gland microbiota. However, most of the genes related to inflammation and apoptosis were expressed at high levels at 72h. Based on the increased mortality of infected oysters under low salinity, it was speculated that the enhanced oyster inflammation would still have adverse effects on oyster health in the later stage. Changes in intestinal microflora are closely related to immune status, as demonstrated in mice infected with *Clostridium difficile* (102). Altered host immune responses to multiple stressors may affect intestinal microbial composition, interspecific interactions, and microbial community mediated biological functions (103). Similarly, studies have shown that *V. anguillarum* infection changes the intestinal flora of *Plecoglossus altivelis*, increasing the abundance of pathogenic bacteria and secreting specific bacterial inducers, which further promotes the expression of immune-related genes (104). The intestinal mucosa also recognized bacterial antigens, which initiated systemic immune-related immune and inflammatory responses (105, 106). Therefore, when the gut flora is impaired, immune homeostasis is also disrupted, which increases the organism’s susceptibility to disease (107). In our study, we also observed alteration of the microbial community structure. The increased abundance of pathogenic bacteria such as *Vibrio* caused intestinal inflammation, while related genes such as immune inflammation were also continuously induced. Therefore, we speculate that low salinity may indirectly affect immune homeostasis by altering the oyster’s digestive bacterial microbiota homeostasis in infected oysters; on the other hand, low salinity may indirectly affect host intestinal microbiota homeostasis by inducing immune changes in infected oysters. At present, in-depth studies on the complex interactions between oyster intestinal microbiome and host immunity are lacking. Future work focusing on this process warrants better understanding of its role in surveilling host health and pathogenesis.

## Conclusion

In this study, we performed an experiment by low salinity stress and pathogen infection with *V. alginolyticus* to investigate their synergistic effect on the mortality of the infected *C. gigas* toward understanding of the interaction among environment, host, and pathogen. The infected oysters exhibited higher mortality rate under low salinity stress compared to normal salinity. Further investigation revealed that high mortality rate was not due to effect of low salinity stress on promoting growth and virulence of *V. alginolyticus*, but likely due to disruption of homeostasis of digestive bacterial microbiota in infected oysters, leading to the burst of pathogenic bacteria as well as excessive inflammatory response to cause immune dysregulation. The interaction of these factors ultimately impairs the oyster’s capacity to defend against infection of *V. alginolyticus* at low salinity, causing mass mortality. Oysters are exposed to various stressors in natural marine environment, so based on the observation of our work, we suggest that abiotic factors such as low salinity and biotic factors such as *Vibrio* pathogens should be considered simultaneously, and the interaction among these stressors should be emphasized to better assess the environmental risk of pathogenic diseases. Moreover, our work revealed that oysters exposed to low salinity stress were more prone to inflammatory responses which benefit infection of virulent pathogens such as *Vibrio* species. Therefore, to vertically move the oysters deeply within the water column by adjusting buoy could be an effective solution to prevent large-scale outbreaks of diseases after extreme precipitation event, which usually causes abrupt decrease of sea surface salinity. Future research deserves further investigations on the relative weight of environmental stressor, host genetics and microbial diversity in the development of oyster disease to identify effective solutions to disease surveillance and control in oyster aquaculture.

## Data Availability Statement

The datasets presented in this study can be found in online repositories. The names of the repository/repositories and accession number(s) can be found below: https://www.ncbi.nlm.nih.gov/sra, accession ID: PRJNA756403 and PRJNA756710.

## Ethics Statement

Experiments related to oysters were carried out in strict accordance with the Management Rule of Laboratory Animals (Chinese Order No. 676 of the State Council, revised 1 March 2017). The Committee on the Ethics of Animal Experiments of Ocean University of China has approved the relevant experimental procedures.

## Author Contributions

SL conceived the study and obtained the funding. XL and SL designed the salinity and *Vibrio* stress related experiment. XL, HW, and RY performed the experiment. XL, BY, and CS analyzed the related data. XL drafted the manuscript and produced figures and tables. SL revised the manuscript. QL supervised the work. All authors contributed to the article and approved the submitted version.

## Funding

This work was funded by the grants from National Natural Science Foundation of China (No. 31802293 and No. 41976098), the Young Talent Program of Ocean University of China (No. 201812013), and the Fundamental Research Funds for the Central Universities (No. 20204201).

## Conflict of Interest

The authors declare that the research was conducted in the absence of any commercial or financial relationships that could be construed as a potential conflict of interest.

## Publisher’s Note

All claims expressed in this article are solely those of the authors and do not necessarily represent those of their affiliated organizations, or those of the publisher, the editors and the reviewers. Any product that may be evaluated in this article, or claim that may be made by its manufacturer, is not guaranteed or endorsed by the publisher.
