# Supplementary figures and images for: Synergistic Interaction of Low Salinity Stress With Vibrio Infection Causes Mass Mortalities in the Oyster by Inducing Host Microflora Imbalance and Immune Dysregulation
Source: Front Immunol. 2022 May 19;13:859975. doi: 10.3389/fimmu.2022.859975 (PMC9162580; doi:10.3389/fimmu.2022.859975)

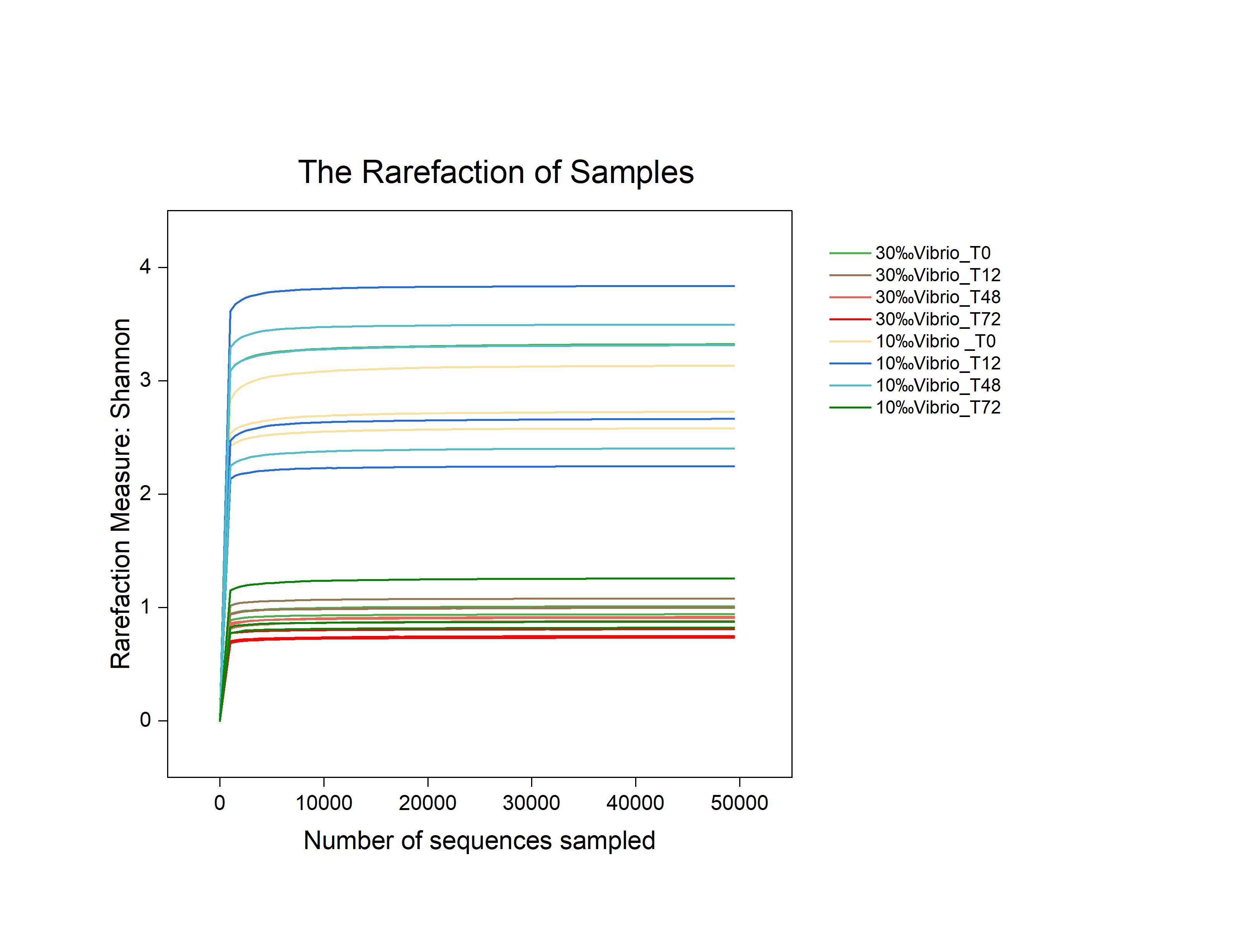

Supplement: Supplementary file 1 [file Image_1.tif]

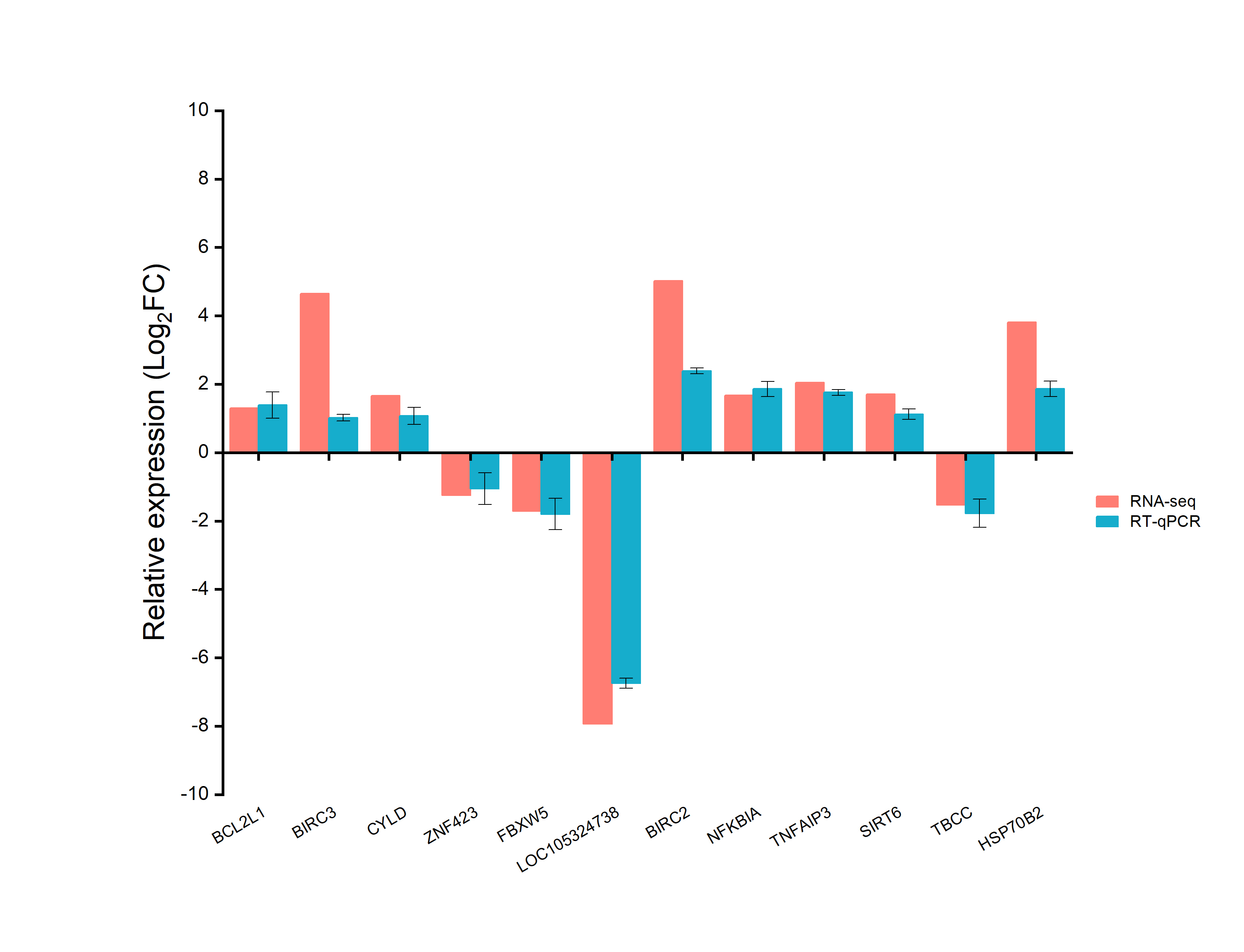

Supplement: Supplementary file 2 [file Image_2.tif]
